# A Case of Aggressive Multiple Myeloma with Extramedullary Involvement of the Female Reproductive System, Thyroid and Breasts

**DOI:** 10.1155/2019/7348504

**Published:** 2019-07-31

**Authors:** Abdul Moiz Khan, Ibrahim Azar, Saleh Najjar, Travis Bevington, Syed Mehdi

**Affiliations:** ^1^Department of Internal Medicine, Albany Medical College, Albany, NY, USA; ^2^Department of Pathology, Albany Medical College, Albany, NY, USA; ^3^Department of Radiology, Albany Medical College, Albany, NY, USA; ^4^Section of Hematology/Oncology, Stratton VA Medical Center, Albany, NY, USA

## Abstract

Extramedullary disease at the time of diagnosis of multiple myeloma is a rare finding that portends poor prognosis and necessitates aggressive treatment strategies. We present a case of multiple myeloma with extramedullary plasmacytomas of the female reproductive system, thyroid and breasts. The patient was treated with lenalidomide, bortezomib, cyclophosphamide, and dexamethasone. Follow-up PET-CT scans confirmed clinical complete response, and the patient underwent autologous stem cell transplantation. The patient will be continued on lenalidomide and bortezomib maintenance therapy. To the best of our knowledge, simultaneous involvement of these sites has never been reported. The case highlights that there are no established guidelines on the treatment of multiple myeloma with extramedullary disease leading to great variability based on clinician preference. We will also discuss the treatment options and prognosis of multiple myeloma with extramedullary disease.

## 1. Introduction

Multiple myeloma (MM) is a disorder of clonal proliferation of plasma cells. MM is defined as clonal bone marrow plasma cells ≥10% or biopsy-proven bony or extramedullary plasmacytoma, and one or more myeloma-defining features (hypercalcemia, renal failure, anemia, lytic bone lesions, ≥60% clonal bone marrow plasma cells, and serum-free light chain ratio ≥100) [1]. It constitutes 10–13% of all hematologic malignancies [2, 3] with an age-adjusted incidence rate of about 4–6 per 100,000 persons per year [2, 4]. Extramedullary disease (EMD) at the time of diagnosis of MM is rare (0.4% to 2%) [5, 6], but during the course of the disease, as many as 13% to 30% of the patients may exhibit extramedullary involvement [7, 8]. The most common extramedullary sites of plasmacytosis are the upper respiratory tract and sinonasal region [9]. However, on reviewing the literature, it is evident that virtually any organ can be affected [7, 10]. Involvement of multiple organs with EMD at the same time is extremely unusual. Of note, plasma cell disorders involving the female reproductive system, thyroid and breasts, are exceptionally uncommon.

Here, we present a case of multiple myeloma with extramedullary plasmacytomas of the female reproductive system, thyroid gland and breasts.

## 2. Case

A 41-year-old female presented with pelvic pain, profuse menorrhagia, and severe symptomatic anemia (near syncope and shortness of breath). She underwent total abdominal hysterectomy with bilateral salpingo-oophorectomy (TAH/BSO) as the continuous bleeding was not responding to conservative measures and the patient had no further desire of fertility. Biopsy showed an infiltrate in the endomyometrium, fallopian tubes, and cervix of mature-appearing plasma cells that stained positive for CD56, CD38, CD79A, and MUM1 with lambda light chain restriction, consistent with plasmacytosis. Bone marrow (BM) biopsy demonstrated a hypercellular marrow and atypical plasmacytosis (25%) with lambda restriction. BM flow cytometry was positive for CD38, CD138, and CD19 and negative for CD56, CD20, and CD45 consistent with plasma cell neoplasm. FISH showed 46, [XX], female karyotype, positive for three copies of 1q21 including CKS1B gene. Free kappa and lambda light chain levels in urine were 39.9 (reference range 1.35–24.19) and 1.59 (0.24–6.66) with a kappa/lambda ratio of 25.09 (2.04–10.37). Free kappa and lambda light chains in serum were 12.6 (3.3–19.4) and 16.5 (5.7–26.3) with a kappa/lambda ratio of .76 (0.26–1.65). No M protein was detected in the SPEP or UPEP. The albumin level was 3.6, beta-2 microglobulin was 1.347 mg/L, and LDH was 163. The patient was diagnosed with nonsecretory multiple myeloma with extramedullary plasmacytoma (ISS stage I).

**Figure 1:**
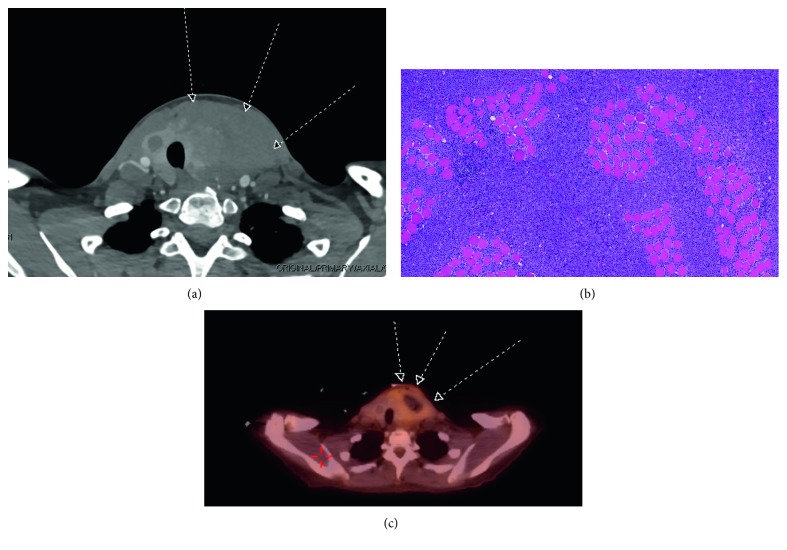
(a) CT scan showing thyroid gland with large left lobe mass. (b) Sheets of monoclonal plasma cells infiltrating skeletal muscle fibers, H&E. (c) PET-CT showing 4.4 × 4.7 cm mass with central necrosis within the left thyroid lobe.

Around the time of TAH/BSO, the patient also reported progressively painful swelling on the left side of her neck accompanied with odynophagia, dysphagia, and hoarseness. CT of neck showed a left thyroid mass (7 × 5 × 7 cm) with irregular borders and a multinodular thyroid enlargement. (Figure 1(a)). Diagnostic incision biopsy demonstrated a very friable, irregular, malignant-appearing mass in the left lobe. A portion of the mass was removed and sent for analysis. Biopsy confirmed lambda-restricted plasma cell neoplasm involving the strap muscles (Figure 1(b)). The neoplastic plasma cells were positive for CD138, CD79a, CD45 (dim), MUM1, VS38C, and CD56. The patient remained clinically and biochemically euthyroid.

**Figure 2:**
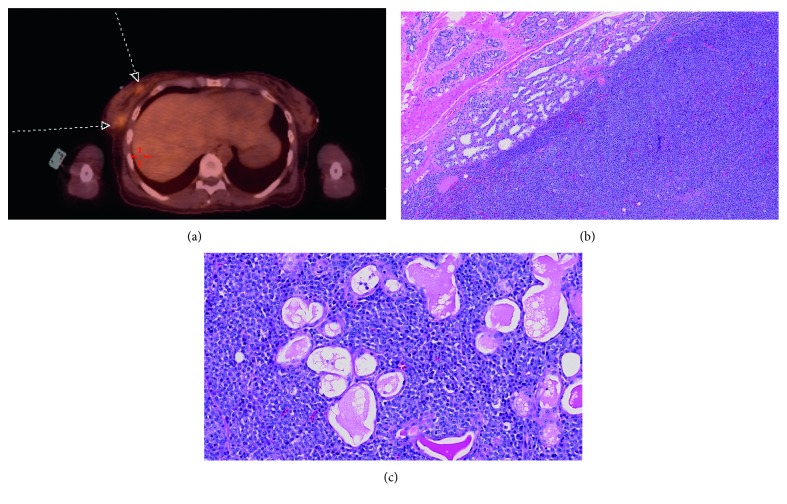
(a) PET-CT showing right breast nodules with FDG uptake. (b) Breast lesion consisting of monoclonal plasma cells, H&E. (c) Monoclonal plasma cells permeate the native breast lobules, H&E.

Whole body FDG-PET scan showed FDG uptake in a 4.4 × 4.7 cm mass with central necrosis within the left thyroid lobe (Figure 1(c)) along with mild FDG uptake within two right breast nodules. (Figure 2(a)). Breast U/S and mammography confirmed the nodules on the PET scan and showed other multiple bilateral nodules. Excision biopsy of the right breast nodule also showed lambda-restricted plasma cell neoplasm (Figure 2(b) and 2(c)) positive for CD 56, CD138, CD79a, and VS38C. The patient also started complaining of lower extremity bone pain, but skeletal survey was negative.

The patient was started on bortezomib (Velcade), cyclophosphamide, and dexamethasone (VCD). After the first cycle, lenalidomide (Revlimid) was added to the regimen (RVCD). The patient completed a total of 1 cycle of VCD and 4 cycles of RVCD. Follow-up PET-CT scan done 6 weeks after the 5th cycle showed no evidence of active lesions. The patient had a clinical complete response (CR), so she proceeded with autologous stem cell transplantation (ASCT) 8 weeks after the 5th cycle of chemotherapy with high-dose melphalan conditioning. The patient tolerated the treatment well. She will be continued indefinitely on daily lenalidomide and alternate weekly bortezomib maintenance therapy and will be followed up with PET-CT scans.

## 3. Discussion

This case poses multiple challenges right from its presentation to its treatment. First, profuse uterine bleeding is an extremely rare mode of initial presentation of MM [11]. Female reproductive organs, breasts, skeletal muscle, and thyroid gland, are exceedingly rare sites of EMD reported mostly in isolated case reports [11–13]. To the best of our knowledge, this is the only case report of MM with simultaneous involvement of the female reproductive system, thyroid and breasts.

Based on our current knowledge, multiple myeloma can lead to formation of soft tissue plasmacytomas most commonly from direct extension and local invasion of skeletal lesions. Rarely, MM can spread through hematogenous dissemination. Molecular mechanisms of EMD are poorly understood. However, reduced expression of cell adhesion molecules like VLA-4, CD44, P-selectin, and CD56, promotion of homing of myeloma cells by decreased expression of chemokine receptors like CCR1, CCR2, and CXCR4, and increased angiogenesis have been proposed as mechanisms [14]. In our case, the absence of bony lesions and the distance between sites of plasmacytosis points towards hematogenous spread.

Multiple studies have shown that extramedullary disease presages a poor prognosis and warrants more aggressive treatment strategies. A study of 1,003 myeloma cases by Varettoni et al. showed that EMD had an adverse prognostic impact on overall survival (hazard ratio 3.5) and progression-free survival (H.R 1.5), even more so when present at the time of diagnosis [7]. Usmani et al. also found that the 5-year overall survival in patients with extramedullary disease at diagnosis was shorter than in non-EMD patients (31% versus 59%). Presence of EMD at diagnosis was also associated with poor prognostic factors at baseline like anemia, thrombocytopenia, elevated centrosome index, and unfavorable cytogenetic abnormalities like MF molecular subtype (with translocation 14; 16 or 14; 20) and PR molecular subtype [15]. Our patient was positive for three copies of 1q21 with CKS1 gene which confers intermediate to high risk. An extra copy of 1q21 (CKS1B) is considered to be an adverse prognostic indicator. Gain/amplification of 1q21 increases the risk of MM progression, and incidence of amplification is higher in relapsed MM than in newly diagnosed patients [1, 16]. No other chromosomal abnormalities were found.

There are currently no established guidelines for the treatment of multiple myeloma with EMD. However, our current understanding is that patients with extramedullary myeloma eligible for stem cell transplantation could be treated with a triplet induction therapy followed by autologous stem cell transplantation (ASCT), triplet consolidation therapy, and maintenance with lenalidomide at least. For elderly patients not eligible for stem cell transplantation, bortezomib-melphalan-prednisone (VMP) or continuous lenalidomide-dexamethasone (Len-Dex) therapy may be done [1, 3, 16, 17]. Another treatment approach for aggressive disease with multiple plasmacytomas may be regimens like VDT-PACE (bortezomib, dexamethasone, thalidomide, cisplatin, doxorubicin, cyclophosphamide, and etoposide). ASCT may be done afterwards if eligible, and maintenance with bortezomib-based regimen will be done [1, 3, 16, 17] (see Table 1 for summary). In our case, given the aggressiveness of disease and atypical sites of involvement, a four-drug regimen (RVCD) was used, and maintenance therapy with both lenalidomide and bortezomib will be done. VDT-PACE regimen was a consideration, but our patient achieved a complete clinical response with RVCD therefore obviating its need.

As for the drugs used for treatment of MM with EMD, bortezomib has proven efficacy and may be incorporated in the multitude of treatment regimens [18]. Conversely, studies have mounted evidence against thalidomide's efficacy in treating multiple myeloma with EMD [19]. Emerging drugs include the immunomodulatory drug pomalidomide which is shown to be efficacious even against the extramedullary component [6]. Other myeloma drugs like daratumumab, elotuzumab, and carfilzomib are also being studied for their role in MM with EMD [3]. As far as the role of autologous hematopoietic stem cell transplantation (ASCT) is concerned, it provides survival advantages but is less efficacious in patients with extramedullary disease [10, 20]. In addition, with EMD, tandem ASCT does not give any survival benefit over single transplantation [21].

## 4. Conclusions

Our discussion should underscore the fact that there is a dearth of prospective studies and no unanimous guidelines regarding the treatment of multiple myeloma with extramedullary disease. So, there might be great subjectivity and variability based on clinician preference. With the advent of new treatment options, the need for more studies becomes even more pertinent to ensure an evidence-based approach.

## Figures and Tables

**Table 1:** Treatment regimens that can be used in multiple myeloma with extramedullary disease.

Stem cell transplantation eligible patients	Triplet induction therapy followed by ASCT, triplet consolidation therapy, and maintenance with lenalidomide at least
Elderly patients not eligible for stem cell transplantation	Bortezomib-melphalan-prednisone (VMP) or continuous lenalidomide-dexamethasone (Len-Dex)
Very aggressive disease especially with multiple plasmacytomas	VDT-PACE (bortezomib, dexamethasone, thalidomide, cisplatin, doxorubicin, cyclophosphamide, and etoposide) followed by ASCT if eligible and maintenance with bortezomib-based regimen
Treatment modalities under study which may be used with traditional regimens	Pomalidomide, daratumumab, elotuzumab, carfilzomib

## Abbreviations

MM: Multiple myeloma
EMD: Extramedullary disease
TAH/BSO: Total abdominal hysterectomy with bilateral salpingo-ophorectomy
ASCT: Autologous stem cell transplantation.

## References

[1] Rajkumar S. V. (2018). Multiple myeloma: 2018 update on diagnosis, risk-stratification, and management. *American Journal of Hematology*.

[2] Rajkumar S. V., Dimopoulos M. A., Palumbo A. (2014). International myeloma working group updated criteria for the diagnosis of multiple myeloma. *The Lancet Oncology*.

[3] Touzeau C., Moreau P. (2016). How I treat extramedullary myeloma. *Blood*.

[4] Kyle R. A., Therneau T. M., Rajkumar S. V., Larson D. R., Plevak M. F., Melton L. J. (2004). Incidence of multiple myeloma in Olmsted county, Minnesota. *Cancer*.

[5] Kyle R. A., Gertz M. A., Witzig T. E. (2003). Review of 1027 patients with newly diagnosed multiple myeloma. *Mayo Clinic Proceedings*.

[6] Short K. D., Rajkumar S. V., Larson D. (2011). Incidence of extramedullary disease in patients with multiple myeloma in the era of novel therapy, and the activity of pomalidomide on extramedullary myeloma. *Leukemia*.

[7] Varettoni M., Corso A., Pica G., Mangiacavalli S., Pascutto C., Lazzarino M. (2010). Incidence, presenting features and outcome of extramedullary disease in multiple myeloma: a longitudinal study on 1003 consecutive patients. *Annals of Oncology*.

[8] Varga C., Xie W., Laubach J. (2015). Development of extramedullary myeloma in the era of novel agents: no evidence of increased risk with lenalidomide-bortezomib combinations. *British Journal of Haematology*.

[9] Ge S., Zhu G., Yi Y. (2018). Extramedullary plasmacytoma of the larynx: literature review and report of a case who subsequently developed acute myeloid leukemia. *Oncology Letters*.

[10] Weinstock M., Aljawai Y., Morgan E. A. (2015). Incidence and clinical features of extramedullary multiple myeloma in patients who underwent stem cell transplantation. *British Journal of Haematology*.

[11] Feldman A. M., Zhang Z., Buekers T., Elshaikh M. A. (2017). Management of gynaecologic plasmacytoma: a review article. *Journal of Obstetrics and Gynaecology*.

[12] Surov A., Holzhausen H. J., Ruschke K., Arnold D., Spielmann R. P. (2010). Breast plasmacytoma. *Acta Radiologica*.

[13] Hassan M. J., Khans S., Pujani M., Jetley S., Raina P. K., Ahmad R. (2014). Extramedullary plasmacytoma of the thyroid: report of a rare case. *Blood Research*.

[14] Bladé J., Fernández de Larrea C., Rosiñol L., Cibeira M. T., Jiménez R., Powles R. (2011). Soft-tissue plasmacytomas in multiple myeloma: incidence, mechanisms of extramedullary spread, and treatment approach. *Journal of Clinical Oncology*.

[15] Usmani S. Z., Heuck C., Mitchell A. (2012). Extramedullary disease portends poor prognosis in multiple myeloma and is over-represented in high-risk disease even in the era of novel agents. *Haematologica*.

[16] (2018). Multiple myeloma- NCCN evidence blocks. https://www.nccn.org/professionals/physician_gls/pdf/myeloma_blocks.pdf.

[17] Bladé J. (2015). How I treat refractory disease and extramedullary disease. *Clinical Lymphoma Myeloma and Leukemia*.

[18] Kapoor P., Ramakrishnan V., Rajkumar S. V. (2012). Bortezomib combination therapy in multiple myeloma. *Seminars in Hematology*.

[19] Rosiñol L., Cibeira M. T., Bladé J (2004). Extramedullary multiple myeloma escapes the effect of thalidomide. *Haematologica*.

[20] Kumar L., Gogi R., Patel A. K. (2017). Multiple myeloma with extramedullary disease: impact of autologous stem cell transplantation on outcome. *Bone Marrow Transplantation*.

[21] Gagelmann N., Eikema D.-J., Iacobelli S. (2018). Impact of extramedullary disease in patients with newly diagnosed multiple myeloma undergoing autologous stem cell transplantation: a study from the Chronic Malignancies Working Party of the EBMT. *Haematologica*.

